# Ultrasound‐guided sampling of the lateral abdominal vein in the grey nurse shark (*Carcharias taurus*, Rafinesque 1810)

**DOI:** 10.1002/vms3.272

**Published:** 2020-04-26

**Authors:** Nicholas Otway

**Affiliations:** ^1^ New South Wales Department of Primary Industries Port Stephens Fisheries Institute Taylors Beach Australia

**Keywords:** biochemistry, blood, elasmobranch, phlebotomy, serum

## Abstract

The paired lateral abdominal veins (LAV) provide alternative venipuncture sites in grey nurse sharks (*Carcharias taurus*, Rafinesque 1810) and their efficacy was assessed using morphometrics, necropsies, ultrasound‐guided blood sampling and by comparing serum biochemistry between the LAV and caudal vein (CV) with values from the latter published previously. The mean length of the CV was 2.8% of total length (TL) whereas each LAV was 22.4% of TL and, when combined, was approximately 16 times longer than the CV. The mean tissue thickness overlying each LAV increased significantly (*p* < .001) with increasing TL and ranged from 3.5 to 33.8 mm in the smallest to largest shark. The mean internal diameter of the paired LAV also increased significantly (*p* < .001) with increasing TL and was equal to or exceeded the CV. Experienced SCUBA divers captured 56 free‐living grey nurse sharks and 46 healthy animals were sampled for blood from the LAV and CV with minimal risk to the animals or staff. Venipuncture of the LAV (*n* = 16) was easily accomplished using ultrasound guidance with a 38 mm/18‐gauge needle, whereas standard methods were used with the CV (*n* = 30). Serum biochemistry was compared (*t*‐tests) and none of the biochemical analytes differed significantly between the LAV and CV. The paired LAV produced representative blood samples and could also be used for fluid therapy and/or intravenous anaesthesia as has been done in other sharks. It is recommended that veterinary/husbandry staff familiarize themselves with the paired LAV and consider their use in the future.

## Graphical abstract

Ultrasound‐guided sampling of blood from the lateral abdominal veins and comparison of serum biochemistry with the caudal vein indicates that the lateral abdominal veins provide efficacious alternatives for venipuncture in grey nurse sharks.

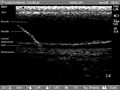

## 1 INTRODUCTION

Blood sampling from the caudal vein (CV) in sharks was first described by Stoskopf, Smith, and Klay (1984) and has been used in studies on shark health and husbandry, reproductive endocrinology and animal transport (e.g. Anderson, Huber, & Berzins, 2012; Henningsen, Murru, Rasmussen, Whitaker, & Violetta, 2008; Smith, 1992; Stoskopf, 2010). The CV is a relatively short vessel located in the haemal arch of the caudal peduncle vertebrae (Muñoz‐Chápuli, 1999) with blood‐flow dependent on the caudal fin muscle contractions involved with swimming (Satchell, 1999). This results in reduced flows during periods of inactivity and, more importantly, when restrained for blood sampling. Needle deviation during caudal venipuncture can result in sampling failures while the occlusion of the needle by cartilage and/or inappropriate caudal fin elevation (Stamper, 2004; Stoskopf et al., 1984) can lead to small blood volumes. While styletted spinal needles can prevent needle occlusion, they can leave large, exit wounds and when combined with the reduced contractibility of shark skin (Stamper, 2004), their use in small individuals can be problematic. Consequently, other venipuncture sites including the heart, caudal artery and the dorsal fin blood sinus (Cliff & Thurman, 1984; Mylniczenko, Curtis, Wilborn, & Young, 2006; Naples, Mylniczenko, Zachariah, Wilborn, & Young, 2012) have been used for blood sampling and differences in serum analyte values between venipuncture sites have been evident (Naples et al., 2012). Nevertheless, the CV has been the preferred vessel for blood sampling with numerous captive and free‐living sharks including the grey nurse shark, *Carcharias taurus*, Rafinesque 1810.

The grey nurse shark in Australian aquaria is synonymous with the sand tiger and ragged‐tooth sharks in similar facilities in the USA, Europe, South Africa and Asia. The shark readily adapts to captivity (Bass, D’Aubrey, & Kistnasamy, 1975; Dehart, 2004) and its large size, generally placid temperament, and continuous display of awl‐like teeth makes it one of the commonest and sought‐after sharks in aquaria worldwide (AES, 2008; Dehart, 2004). In the wild, the disjunct grey nurse shark populations are listed globally as “Vulnerable” on the International Union for the Conservation of Nature (IUCN) “Red List” because of fishing‐related interactions (Otway, Bradshaw, & Harcourt, et al., 2004; Otway, 2015). More recently, research on the “critically endangered” grey nurse shark population off eastern Australia has enabled the collection of blood samples via the CV from free‐living individuals providing important data for husbandry and veterinary care (Otway, 2015). This ongoing research also provided an opportunity to assess the efficacy of an alternative venipuncture site provided by the paired lateral abdominal veins (LAV).

The paired LAV originate in the cloacal region and run cranially from the pelvic to pectoral fins along the lateroventral muscle mass adjacent to the peritoneum (Muñoz‐Chápuli, 1999; Satchell, 1999). Blood‐flow in the paired LAV is driven by the ventricular ejection of blood from the heart (Satchell, 1999) and produces flows that are not greatly affected by inactivity or restraint. The precise location of the paired LAV cannot be easily identified using external morphological features and venipuncture requires ultrasound guidance. With this in mind, an assessment of the LAV was done by integrating the results of morphometric measurements, necropsies, ultrasound‐guided blood sampling and by comparing the serum biochemistry obtained from the LAV with those obtained previously from the CV by Otway (2015).

## 2 MATERIALS AND METHODS

### 2.1 Necropsies

As part of a multi‐disciplinary threatened species recovery program, 190 grey nurse sharks from the wild population of eastern Australia have been subjected to necropsy following their incidental capture in shark nets protecting swimmers at beaches and by commercial and/or recreational fishers. However, quantitative necropsy data for this study were only taken from 64 sharks with no evidence of previous fishing interactions (e.g., hook retention in the gastrointestinal tract) prior to their death. These individuals comprised neonates to sexually mature individuals of both sexes and the necropsies were used to confirm the location of the paired LAV and CV, their anatomy and surrounding tissues/organs. The total length (TL) with caudal fin in a depressed position (Francis, 2006) and total weight (TW) were recorded to the nearest mm and kg, respectively, for sharks ranging 1000–3050 mm TL. Fifty additional measurements including the pre‐caudal length (PCL); pectoral‐pelvic space—the straight‐line length between the pectoral fin insertion and the pelvic fin origin (PPS); the girth at the cranial edge of the pre‐caudal pit (G_PCP_) and the anal‐caudal space—the straight‐line length between the anal fin insertion and the origin of lower lobe of the caudal fin (ACS) were also taken and plotted against TL.

**Figure 1:**
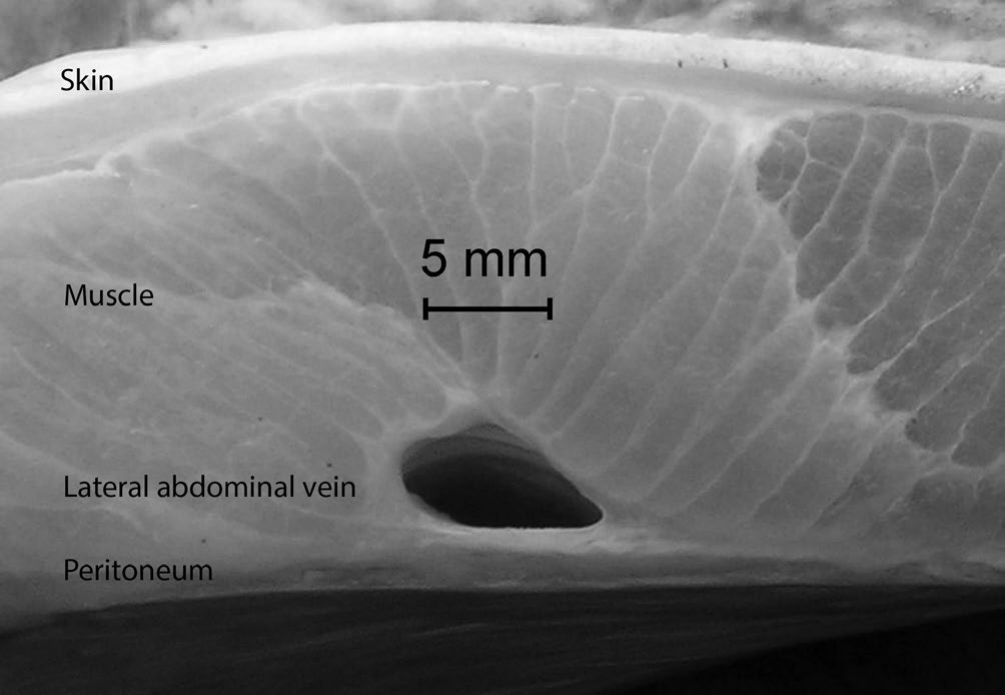
Photograph of transverse section of the abdominal wall of a grey nurse shark (*Carcharias taurus*) showing the skin, muscle, lateral abdominal vein and peritoneum

The ultrasonographic depth of focus to the LAV and length of needle required were identified by quantifying the mean tissue thickness (MTT) overlying each LAV and the mean internal diameter (MID) of the paired LAV. Both variables were measured to the nearest 0.1 mm using digital photographs of transverse sections with a 30 mm scale bar (Figure 1) at four equidistant positions along the paired LAV and measurement software (GRAB IT XP, Datatrend Software).

### 2.2 Sampling sites, shark capture and blood sampling

To permit comparison of blood samples from the same (reference) population, free‐living grey nurse sharks occupying sand and/or boulder‐filled gutters close to islands and emergent rocky pinnacles (Otway & Ellis, 2011) were captured from small dive boats (< 7m) on days with low seas and swell and a minimal sea‐breeze. In addition to the 37 grey nurse sharks (19 males, 18 females) previously sampled by Otway (2015), a further 19 (9 males, 10 females) were captured by a team of experienced scuba divers using identical methods that involved placing a lasso around the caudal peduncle of each shark. Following capture, each shark was hauled to the surface, placed in a partially submerged stretcher and rolled into dorsal recumbency to induce tonic immobility (Henningsen, 1994). Venipuncture was given chronological primacy over the health assessment to reduce the effects of capture stress on serum biochemistry (see Otway, 2015 for details) and blood was collected from every shark captured.

**Figure 2:**
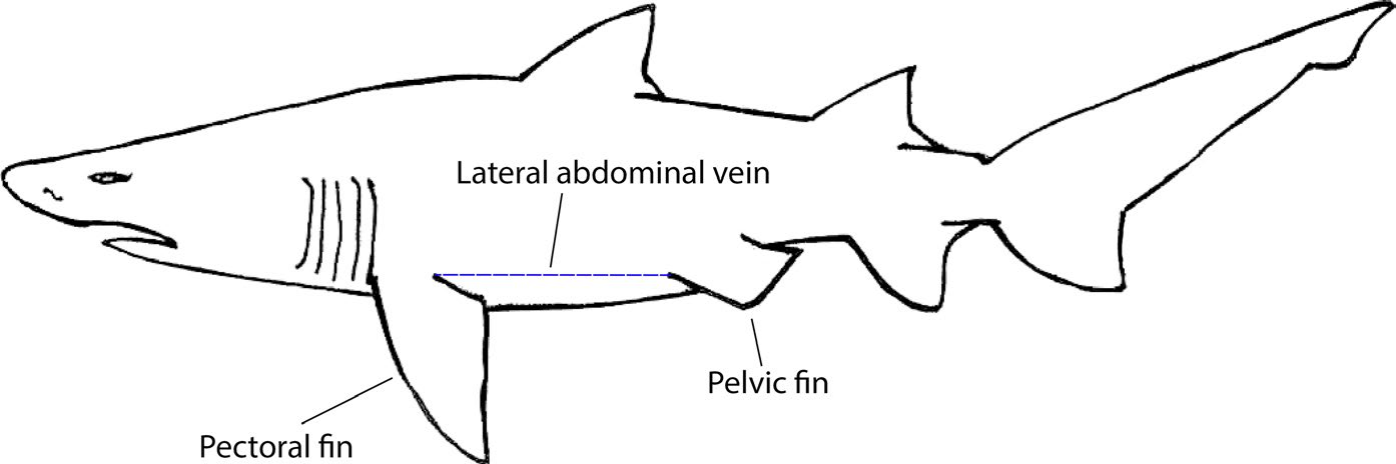
Diagram showing location of the lateral abdominal vein in relation to the pectoral and pelvic fins in the grey nurse shark (*Carcharias taurus*)

A portable Sonosite TITAN (Sonosite, Sydney, Australia) colour, doppler ultrasound system with a 5–10 MHz, 38 mm linear array transducer (7 cm maximum scan depth) was used for ultrasound guidance. Acoustic coupling gel (Image Gel 200—ATX Medical Solutions, Sydney, Australia) was applied liberally to the shark's skin 50–100 mm cranial to the left or right pelvic fin origin (Figure 2). The ultrasound transducer (orientated transversely) was placed on the coupling gel and a preliminary scan was done to confirm the local anatomy comprising the skin, muscular abdominal wall, the LAV, peritoneum and liver. The portable ultrasound with transducer (orientated longitudinally) along the LAV was then used to guide a disposable syringe with 38 mm, 18 G needle (Terumo) to the LAV.

### 2.3 Health assessment

The tripartite health assessment (Otway, 2015) was done to identify any unhealthy sharks and exclude their sera from analysis in this study. Firstly, the presence/absence of buccal (respiratory) pumping, eye appearance, pupillary reflexes, teeth and gingival appearance, skin colour and gender (via claspers in males) were recorded. Secondly, a detailed physical examination was done in which scars, open wounds, external parasites (and associated lesions), muscle tone, spinal deformities and the presence of hooks in the jaws, gills and buccal cavity were noted. The TL, PCL, PPS and G_PCP_ were then measured to the nearest mm with a flexible measuring tape with the PCL and G_PCP_ each used to estimate TL and TW via the regressions in Table 1. Lastly, the sexual maturity of each shark was assessed using TL, PPS and the absence of uterine hymen on cloacal palpation in females, and the TL, degree of clasper calcification and clasper length in males (Walker, 2005). The presence of hooks in the gastrointestinal tract and associated organs was determined using the metal detector and/or ultrasound. Age was then estimated using TL and a von Bertalanfy growth curve. On conclusion, a tissue sample (fin‐clip) was taken and preserved in 95% ethanol for subsequent genetic analysis (e.g. Stow et al., 2006). The grey nurse shark was then released with its subsequent swimming behaviour monitored for 15 min by scuba divers.

**TABLE 1:** Morphometric relationships derived for grey nurse sharks (*Carcharias taurus*) from coastal waters off eastern Australia

Morphometric relationship	Units	Regression equation	Regression significance			
			*n*	*r* ^2^	*F*	*p*
Total weight on Total length	kg, m	TW = 5.4511(TL^3.1716^)	64	0.97	1983.6	<.001
Total weight on Girth at Precaudal pit	kg, m	TW = 2,514.3(G_PCP_^3.9139^)	64	0.99	9,863.0	<.001
Total length on Precaudal length	m, m	TL = 1.3682(PCL) + 0.0685	64	0.99	6,459.0	<.001
Pectoral‐pelvic space on Total length	m, m	PPS = 0.224(TL) + 0.601	47	0.91	520.7	<.001
Anal‐caudal space on Total length	mm, mm	ACS = 0.035(TL) – 14.618	47	0.91	526.9	<.001
Mean tissue thickness overlying LAV on Total length	mm, mm	MTT = 0.015(TL) – 14.303	47	0.90	428.0	<.001
Mean internal diameter of LAV on Total length	mm, mm	MID = 0.0046(TL) – 3.506	47	0.90	426.8	<.001

Abbreviations: ACS, anal‐caudal space; G_PCP_, girth at precaudal pit; LAV, lateral abdominal vein; MID, mean internal diameter of LAV; MTT, mean tissue thickness; PCL, precaudal length; PPS, pectoral‐pelvic space; TL, total length; TW, total weight.

### 2.4 Serum biochemistry

Following venipuncture, the blood was immediately processed using the methods of Otway (2015). Briefly, the blood was placed into a disposable falcon tube without anticoagulant, stored in darkness and on ice until returning to shore. Once ashore and following clotting, the blood was centrifuged at 3,000 *g* for 6 min and then the separated serum was pipetted (1.5 ml) into 3 or 4 replicate capped plastic vials and frozen at −20°C overnight. Thereafter, the sera were transported to the analytical laboratory (IDEXX) and stored at −20°C until analysed (within 3 days). Serum biochemical analytes were quantified via ion selective electrode methods and automated bi‐chromatic spectrophotometry using the same analytical equipment in Otway (2015).

### 2.5 Statistical analyses

Statistical analyses were done with a nominal Type I (α) error‐rate of *p* = .05 using DATADESK Version 6.0 (Data Description Inc.), POPTOOLS (Hood, 2010) and SIGMA PLOT Version 11.0 (Systat Sofware Inc.). TW was plotted against TL and G_PCP_ with curvilinear regressions fitted and the significance examined using analysis of variance (ANOVA). The PCL, PPS, ACS, MTT and MID were also plotted against TL and the respective lines of best fit was calculated using least‐squares linear regression and their significance was examined using ANOVA. Possible differences in the TL or TW of sharks chosen for sampling the LAV or CV and between the sexes were examined using two‐factor ANOVA following Cochran's test for heteroscedasticity and subsequent transformation if required (Underwood, 1997). The proportions of sharks chosen for blood sampling from the LAV and CV (i.e., venipuncture sites) between the sexes or immature and mature individuals were examined using χ^2^ contingency table analyses. The serum analyte data were examined following standard procedures (Otway, 2015). Briefly, the raw data for each serum analyte were plotted and examined for outliers using Tukey's method (Sokal & Rohlf, 1969), with an *a priori* emphasis on retaining all but the most extreme outliers in accordance with previous studies (e.g. Geffré et al., 2009). Shapiro‐Wilk tests were used to examine for normality and unbalanced, 2‐tailed *t*‐tests were used to test the null hypothesis of no difference in mean values between venipuncture sites for each serum analyte.

## 3 RESULTS

### 3.1 Necropsies

A subset of the morphometric relationships of direct relevance to this study are summarized in Table 1. All the linear regressions on TL were significant (*p* < .001) and accounted for at least 90% of the variation in each dependent variable. The PPS closely approximated the position of each LAV and was used to quantify the length of the LAV accessible for venipuncture. The PPS increased significantly with increasing TL (Table 1) and the mean (±*SD*) length of each LAV was 22.4 (0.1) % of TL and ranged from 22.2% to 22.7% of TL. The ACS was used to quantify the length of the CV accessible for venipuncture. While the length of the ACS also increased significantly with increasing TL (Table 1), the mean (±*SD*) length of the CV was only 2.8 (0.2) % of TL and ranged from 2.4% to 3.1% of TL.

The thickness of the tissue overlying each LAV (Figure 1) did not differ significantly among the four positions (ANOVA, *p* = .39). As expected, the MTT increased significantly with increasing TL (Table 1) with the mean MTT (±*SD*) ranging from 3.5 (0.6) mm in the smallest shark to 33.8 (3.8) mm in the largest shark. The MID also increased significantly with increasing TL (Table 1) with the mean MID (±*SD*) ranging from 1.8 (0.2) mm to 11.8 (1.0) mm in the smallest to the largest shark, respectively. Finally, the cross‐sectional area of each LAV was equal to or exceeded that of the CV in any given shark.

### 3.2 Shark capture, blood sampling and health assessment

When lassoed each grey nurse shark swam away from the scuba diver and stopped when the rope (to the boat) became taut. Thereafter, the shark turned 180°, attempted to bite its own tail and then hung limply upside down while it was hauled to the surface. Once in the stretcher, it was rolled into dorsal recumbency and became cataleptic within a couple of minutes with minimal manual restraint required. This ensured the safety of all persons involved and reduced the shark's stress associated with capture and handling.

**Figure 3:**
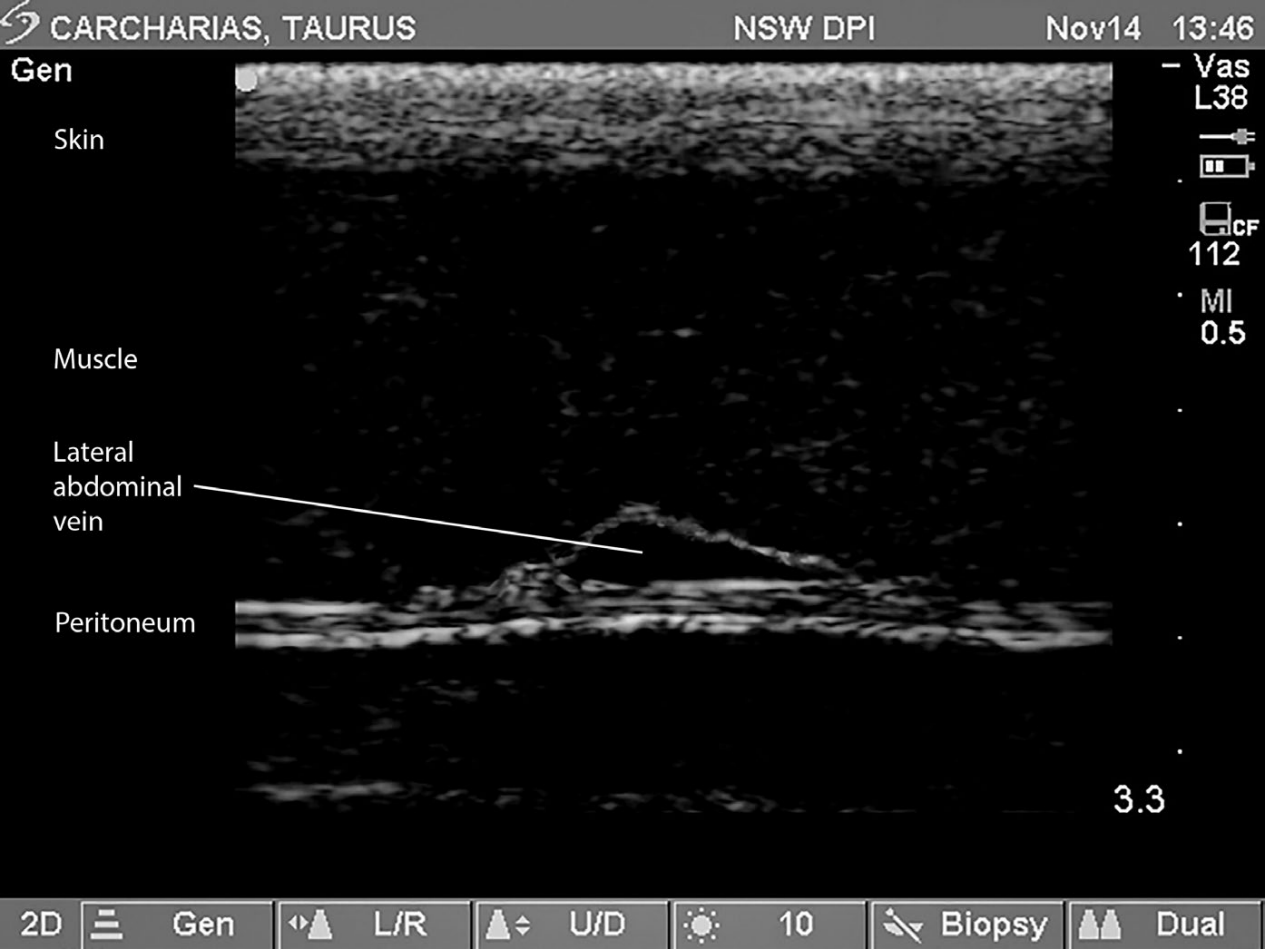
Ultrasound image (transverse section) showing abdominal wall of a 2,600 mm TL male grey nurse shark (*Carcharias taurus*) with skin, muscle, lateral abdominal vein and peritoneum. *Note*: Scale to maximum of 3.3 cm is shown vertically on the right‐hand side of the sonogram with increments of 0.5 cm between markers

**Figure 4:**
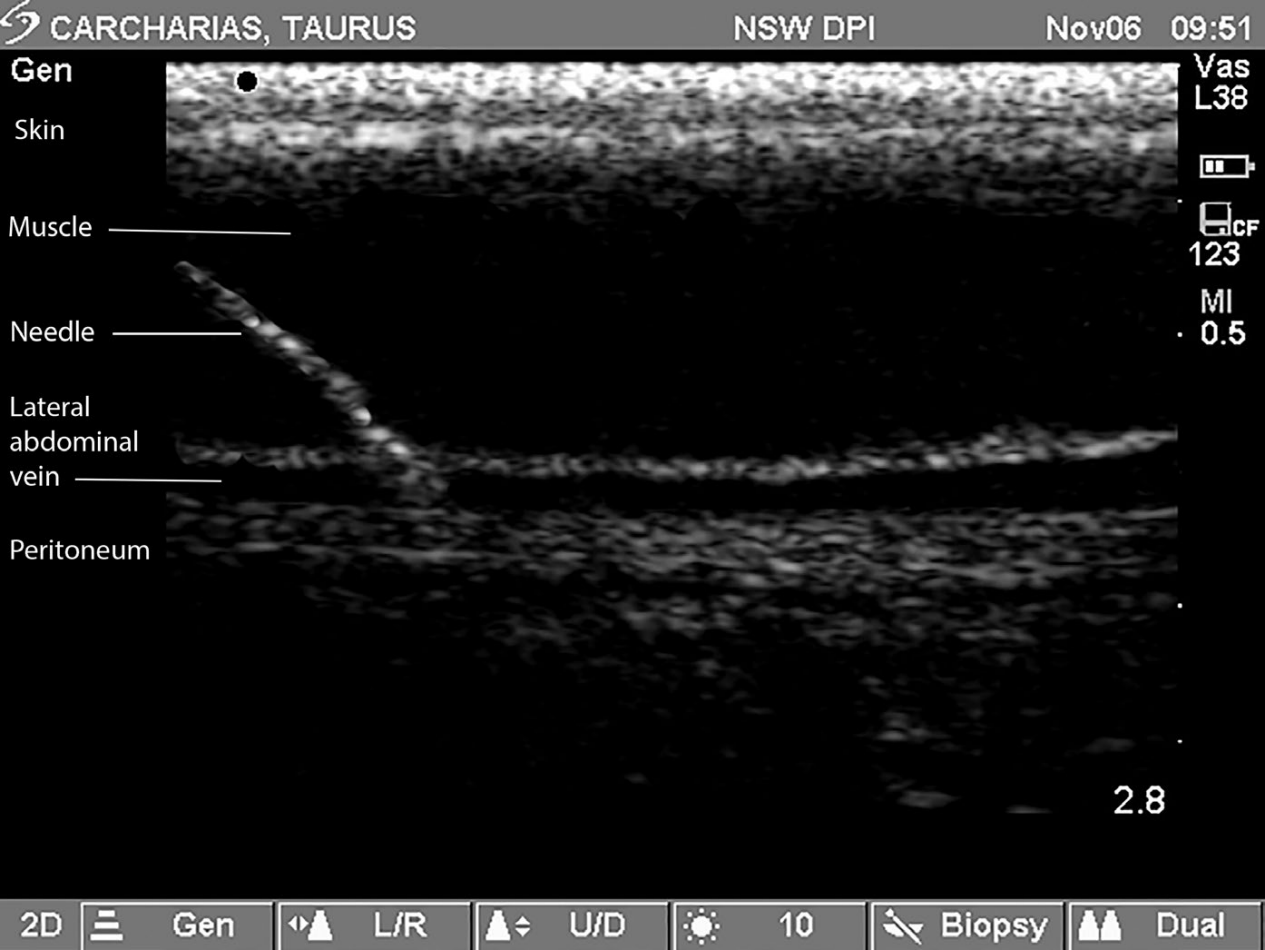
Ultrasound image (longitudinal section) showing abdominal wall of a 2,270 mm TL female grey nurse shark (*Carcharias taurus*) with skin, muscle, needle, lateral abdominal vein and peritoneum. *Note*: Scale to maximum of 2.8 cm is shown vertically on the right‐hand side of the sonogram with increments of 0.5 cm between markers

Ultrasound‐guided blood sampling of the LAV was then easily accomplished with the preliminary scan confirming the local anatomy comprising the skin, muscular abdominal wall, the LAV and peritoneum (Figure 3). A reverberation artefact, manifest as a mirroring of the skin, was evident in the upper 5 mm of some of the ultrasound images (Figure 4) but did not affect the continuous visualization of the needle. The penetration of the LAV was distinctly felt and associated with an instantaneous flash of blood in the syringe nozzle. Ultrasound guidance enabled a single, 10‐mL blood sample to be collected with ease from the left or right LAV in each of the free‐living grey nurse sharks.

Following release, each shark descended to the seabed and rejoined its conspecifics aggregated within a sand or boulder‐filled gutter. Once with the other grey nurse sharks, the released individual resumed its normal swimming behaviour (i.e. ‘hovering’ and/or ‘milling’ sensu Smith, Scarpaci, Louden, & Otway et al., 2015). Moreover, none of the released sharks commenced active buccal pumping (a stress indicator in this species) during the 15‐min monitoring period after blood sampling.

Of the 19 sharks caught for LAV blood sampling, three individuals (one male, two females) with prior fishing‐related injuries were classified as unhealthy using the exclusion criteria of Otway (2015) and eliminated from the study. The remaining individuals (eight males, eight females) were then compared with the 30 healthy sharks caught by Otway (2015) to identify any significant differences in TL, TW and sexual maturity (Table 2). The mean TL and TW of the 46 healthy sharks captured for blood sampling from the LAV and CV (Table 2) did not differ between gender or venipuncture site (ANOVA, *p* = .94 and 0.93, respectively). Similarly, the proportions of immature and sexually mature sharks did not differ significantly between venipuncture site and gender (χ^2^ = 0.05 and 1.39, *p* = .83 and 0.24, respectively).

**TABLE 2:** Comparison of the biological attributes of free‐living grey nurse sharks (*Carcharias taurus*) captured in coastal waters off eastern Australia for sampling blood from the lateral abdominal veins and caudal vein

Venipuncture site	Gender	Number of individuals			Total length (mm)		Total weight (kg)	
		Total	Immature	Mature	Mean (±*SD*)	Range	Mean (±*SD*)	Range
Lateral abdominal vein	Male	8	3	5	2,199 (519)	1,470–2,650	77 (45)	19–120
	Female	8	5	3	2,236 (447)	1,520–2,720	79 (40)	27–130
Caudal vein	Male	15	6	9	2,169 (503)	1,470–2,650	74 (43)	19–120
	Female	15	9	6	2,273 (466)	1,520–2,800	84 (42)	27–143

### 3.3 Serum biochemistry

There was no haemolysis in the serum samples from the LAV and inspection of the two datasets indicated that there were no outliers present (Tukey's method) and the analyte values approximated normal distributions with homogeneous variances (2‐tailed *F*‐tests, all *p* > .05). Moreover, the low serum CK activities (≤81 IU) indicated that the serum biochemistry analytes were unaffected by capture‐stress (Otway, 2015). The mean (±*SD*), median and range for each serum analyte sampled from the LAV and CV were then calculated and summarized in Table 3 together with results of unbalanced, 2‐tailed *t*‐tests which showed that none of the serum biochemistry analytes differed significantly between venipuncture sites (all analytes, *p* = .24–0.98).

**TABLE 3:** Comparison of serum biochemical analytes sampled from the lateral abdominal vein and caudal vein of free‐living grey nurse sharks (*Carcharias taurus*) from coastal waters off eastern Australia

Serum analyte	Lateral abdominal vein (LAV, *n* = 16)			Caudal vein (CV, *n* = 30)			LAV versus CV	
	Mean (±*SD*)	Median	Range	Mean (±*SD*)	Median	Range	*t*	*P*
Sodium (mmol/L)	258.0 (5)	257.0	248.0–267.0	258.0 (4)	257.0	248.0–267.0	0.14	.89
Chloride (mmol/L)	242.0 (8)	246.0	228.0–252.0	242.0 (7)	242.0	228.0–254.0	0.03	.98
Potassium (mmol/L)	4.8 (0.5)	4.8	4.2–5.5	5.0 (0.3)	5.0	4.1–5.5	1.19	.24
Inorganic P (mmol/L)	1.9 (0.1)	1.8	1.7–2.0	1.8 (0.1)	1.9	1.7–2.0	0.43	.67
Total calcium (mmol/L)	3.9 (0.2)	3.9	3.5–4.1	3.9 (0.3)	3.9	3.4–4.3	0.10	.92
Magnesium (mmol/L)	2.0 (0.1)	1.9	1.6–2.2	1.9 (0.2)	1.9	1.6–2.2	0.94	.35
Glucose (mmol/L)	2.7 (0.3)	2.7	2.1–3.0	2.7 (0.2)	2.7	2.1–3.1	0.31	.76
Total protein (g/L)	31.0 (3)	30.0	26.0–36.0	30.0 (3)	30.0	26.0–36.0	0.43	.67
Urea (mmol/L)	376.0 (8)	375.0	365.0–394.0	377.0 (8)	376.0	361.0–394.0	0.19	.92
Creatinine (μmol/L)	30.0 (8)	30.0	20.0–40.0	32.0 (10)	30.0	20.0–40.0	0.58	.57
Total bilirubin (μmol/L)	1.6 (0.5)	2.0	1.0–2.0	1.5 (0.5)	2.0	1.0–2.0	0.30	.77
Cholesterol (mmol/L)	1.4 (0.4)	1.4	0.7–2.1	1.4 (0.3)	1.4	0.9–2.0	0.20	.84
Triglyceride (mmol/L)	0.3 (0.1)	0.3	0.2–0.4	0.3 (0.1)	0.3	0.2–0.5	0.89	.38
ALP (U/L)	21.0 (6)	23.0	10.0–28.0	20.0 (6)	20.0	10.0–28.0	0.78	.44
ALT (U/L)	3.0 (1)	3.0	1.0–3.0	3.0 (1)	3.0	1.0–3.0	0.75	.46
AST (U/L)	30.0 (10)	31.0	15.0–45.0	29.0 (8)	31.0	16.0–43.0	0.51	.61
CK (U/L)	38.0 (20)	33.0	15.0–81.0	42.0 (18)	41.0	17.0–81.0	0.71	.48

Abbreviations; ALP, alkaline phosphatase; ALT, alanine aminotransferase; AST, aspartate aminotransferase; CK, creatine kinase; P, phosphorus.

## 4 DISCUSSION

The necropsies of grey nurse sharks enabled a thorough examination of both blood vessels in neonate to sexually mature individuals of both sexes. The anatomies and morphometric measurements for grey nurse sharks off eastern Australia were similar to those of ragged‐tooth sharks off South Africa (Bass et al., 1975) permitting greater generalization of the results to individuals from other populations. Compared to the CV, the paired LAV received blood from a much larger capillary bed comprising the cloacal region, pelvic girdle, pelvic fins, segmental muscles of the abdominal wall and pectoral girdle (Muñoz‐Chápuli, 1999). When combined, the paired LAV were approximately 16 times longer, on average, than the CV. Additionally, the paired LAV had internal diameters that were equal to or exceeded the CV and venous flows in the LAV are greater and unlikely to be affected by inactivity (Satchell, 1999).

The placement of each shark in dorsal recumbency in a partially submerged stretcher induced catalepsy, reduced the shark's capture and handling stress and ensured the safety of all persons involved. The ultrasound‐guided blood sampling of the LAV was easily accomplished without a styletted needle and at minimal risk to the animal. The reverberation artifact was most likely due to the shield‐shaped denticles in the skin of *C. taurus* (Gilligan & Otway, 2011) but did not affect the visualization of either LAV or the needle. Despite this, the main risk of this procedure is secondary perforation of the LAV with the needle entering the peritoneal cavity. However, three steps can be taken to minimize this risk. First, appropriate restraint of the shark while in dorsal recumbency is necessary to prevent the shark rolling. Second, the MTT overlying each LAV is noted during the preliminary scan. Third, the angle of needle entry under ultrasound guidance is adjusted in relation to the MTT to mitigate secondary perforation of the LAV.

On release, each shark resumed its normal swimming behaviour near the seabed consistent with previous observations at numerous other aggregation sites (Otway, 2015; Smith et al., 2015) and provided strong evidence that the entire procedure had no long‐term adverse effects on this critically endangered species.

The absence of significant differences in the serum biochemistry analytes between the LAV and CV in free‐living grey nurse sharks clearly indicates that either venipuncture site can be used to obtain representative blood samples. The serum biochemistry analytes were also similar to those obtained from the CV in captive grey nurse (sand tiger) sharks in North American aquaria (Anderson et al., 2012). These similar results were somewhat expected as the LAV and CV are part of the primary vascular system in sharks (Muñoz‐Chápuli, 1999; Satchell, 1999). In contrast, the dorsal fin blood sinus forms part of the secondary vascular system in sharks (Muñoz‐Chápuli, 1999; Satchell, 1999) and possible differences between these vascular systems may have contributed to differences in haematocrit and serum analyte values in comparisons with blood drawn from the CV of captive and free‐living sharks in previous studies (Mylniczenko et al., 2006; Naples et al., 2012).

Future use of the paired LAV would enable serial blood sampling of captive sharks to be distributed across two long vessels instead of the single, short CV. The paired LAV may also have substantially greater benefits especially when focusing on small sharks or when other procedures are done. For example, the CV has also been used for the infusion of fluids and/or intravenous anaesthesia (e.g. Lécu, Herbert, Coulier, & Murray, 2011; Smith, 1992; Stamper, 2004 and references therein). Using the paired LAV would also permit fluid infusion, avoiding possible over‐perfusion of the kidneys, and mitigate issues associated with nephrotoxic drugs.

## 5 CONCLUSION

The successful field‐based sampling clearly showed that the entire procedure ensured the safety of all persons involved, had no adverse effects on the sharks and would be easily achieved with captive animals in the sheltered setting of an aquarium. Importantly, the morphometric measurements, necropsies, ultrasound‐guided blood sampling and comparison of serum biochemistry clearly demonstrated that the paired LAV provide an efficacious alternative for venipuncture in grey nurse sharks. With this in mind, aquarium staff responsible for husbandry and veterinary care should take appropriate opportunities (e.g. via necropsies) to familiarize themselves with the paired LAV with a view to their use in the future.

## 6 Ethics Statement

The author confirms that the ethical policies of the journal, as noted on the journal's author guidelines page, have been adhered to. This study was done under a scientific research permit (Permit No. P01/0059[A]) and an Animal Research Authority (99/14 – Port Stephens) following the Australian Government National Health and Medical Research Council Australian code of practice for the care and use of animals for scientific purposes 8th Edition, 2013.

## CONFLICT OF INTEREST

The author declares that there are no conflicts of interest.

## AUTHOR CONTRIBUTION

Nicholas Mark Otway: Conceptualization; Data curation; Formal analysis; Funding acquisition; Investigation; Methodology; Project administration; Resources; Software; Supervision; Validation; Visualization; Writing‐original draft; Writing‐review & editing. 
